# Differentiation of mesenchymal stem cells towards lens epithelial stem cells based on three-dimensional bio-printed matrix

**DOI:** 10.3389/fcell.2024.1526943

**Published:** 2025-01-06

**Authors:** Yufan Liu, Zuowei Wang, Tianju Ma, Yi Gao, Wenqian Chen, Zi Ye, Zhaohui Li

**Affiliations:** Senior Department of Ophthalmology, The Third Medical Center of PLA General Hospital, PLA General Hospital and PLA Medical College, Beijing, China

**Keywords:** MSCs, 3D bio-printed matrix, differentiation, LESCs, crystalline lens

## Abstract

The high risks of traumatic cataract treatments promoted the development of the concept of autologous lens regeneration. Biochemical cues can influence the cellular behavior of stem cells, and in this case, biophysical cues may be the important factors in producing rapid activation of cellular behavior. Here we bio-printed mesenchymal stem cells (MSCs) using a commonly used bioink sodium alginate-gelatin blends, and investigated the induction effect of MSC differentiation towards lens epithelial stem cells (LESCs) under a combination of biochemical cues and biophysical cues. It was found that biochemical cues in the porous three-dimensional (3D) matrix constructed using bioink sodium alginate-gelatin blends for bio-printing did not reduce the cell viability of loaded MSCs in the matrix by scanning electron microscope (SEM) observation and cell viability detection. Loaded MSCs in the matrix were consistently upregulated in the expression of proteins and genes involved in phenotypes and development signaling pathways of LESCs, as detected by polymerase chain reaction (PCR) with the support of biochemical cues. These results indicated that biophysical cues could rapidly activate the cellular behavior of MSCs differentiation, and biochemical cues could continuously induce MSCs differentiation towards LESCs.

## 1 Introduction

Traumatic cataract is a kind of lens opacity caused by various factors of ocular trauma (open or closed), which is also one of the important causes of blindness after ocular trauma (Alfaro et al., 2005; Shah et al., 2008). Traumatic cataract patients are mostly young people, who have higher requirements for postoperative refractive status. Different from simple age-related cataract, traumatic cataract is often accompanied by irreversible damages to ocular structures and acute ocular lesions such as compromised capsular integrity, ruptured suspensory ligament, phaco-anaphylactic uveitis, glaucoma and so on (Vajpayee et al., 1994; Rao et al., 1998; Thomas, 1998; Vajpayee et al., 2001). The irreversible damages to ocular structures and acute ocular lesions will lead to a high risk of surgical treatment or no opportunity for surgical treatment. For patients with traumatic cataract who still have the opportunity of surgical treatment, the current treatment can only be monofocal intraocular lens replacement or monofocal intraocular lens suspension through surgery (Por and Lavin, 2005). A series of disadvantages of intraocular lens suspension include further damage to the ocular structure, long postoperative recovery period, poor visual recovery, and complications such as hemorrhage, intraocular tissue damage, endophthalmitis, intraocular lens rejection, and posterior capsular opacities (PCO) (Li and Jie, 2019; Mohammadpour et al., 2019; Bremond-Gignac et al., 2020). PCO is the most common complication affecting visual recovery after intraocular lens replacement (Wormstone et al., 2009; Fișuș and Findl, 2020). A number of previous studies have shown that the formation of PCO is a manifestation of organ fibrosis, which is induced by tissue damage caused by surgical procedures and inflammatory reaction caused by intraocular lens stimulation. Therefore, the surgical timing and treatment of traumatic cataract are still widely discussed and controversial around the world (Shah et al., 2013). In view of the potential risks of surgical treatment of traumatic cataract, autologous lens regeneration has become the key in the research field of treatment of traumatic cataract. Therefore, the ability of autologous lens regeneration is a potential method to restore the original refractive state of the lens in patients with traumatic cataract. In recent years, the continuous development of stem cell theory has provided new concepts for exploring the regeneration of lens from autologous sources (Filoni, 2009; Stern et al., 2018). Study by Liu et al. have successfully confirmed that endogenous LESCs can be regenerated into lens-like bodies under specific induction protocols (Lin et al., 2016). This result not only confirms the possibility of lens regeneration, but also proves that LESCs is a key point in lens regeneration research. However, the endogenous LESCs used in current research need to be obtained by surgery, which limits the in-depth study of lens regeneration to a certain extent.

Crystalline lens is developed from the ectoderm in the embryo, and the development of lens is inseparable from the activation of a series of signaling pathways. Several studies have shown that FGF, Notch, Wnt and BMP signaling pathways are closely related to the development of crystalline lens (Hayashi et al., 2008; Cvekl and Wang, 2009; Bassnett and Šikić, 2017; Li et al., 2020). Based on the involvement of signaling pathways during lens development, a novel three-stage induction system have been reported. The system was established by adding additional cytokines to induce human embryonic stem cells (hESCs) to differentiate into a large number of lentic-like cells and differentiated 3D lenses (Yang et al., 2010). However, the difficulty of obtaining hESCs and ethical controversy in experimental research have hindered the progress of research to some extent. Therefore, the exploration of the directional differentiation of adult stem cells into LESCs is the key in the study of lens regeneration. Compared with hESCs(Yoshida and Yamanaka, 2017; Gorecka et al., 2019), the application of adult stem cells has become a research hotspot in the field of stem cells due to the advantages of less ethical controversy, wide source and easy access. In recent years, a large number of studies have shown that MSCs play a significant role in tissue and organ regeneration research (Nethi et al., 2023; Li et al., 2024). The proliferation and differentiation of stem cells and the development and regeneration of tissues and organs are inseparable from the 3D environment. Numerous studies have shown that sodium alginate-gelatin composite hydrogel, a well-known bioink for extruded bio-printing, has demonstrated good cell compatibility, printability, and structure retention in long-term culture (Jang et al., 2018; Ong et al., 2018; Yao et al., 2020). Sodium alginate-gelatin composite hydrogel can not only provide a high-quality 3D biomimetic living environment for cells *in vitro*, but also provide physical inducing factors for the directional differentiation of stem cells. A number of previous studies have confirmed that MSCs loaded in sodium alginate-gelatin 3D bio-printing matrix can differentiate into a variety of adult cells under specific biochemical and physical cues, such as bone cells (Pajarinen et al., 2019), adipocytes (Chen et al., 2016), nerve cells (Jin et al., 2019), sweat gland cells (Liu et al., 2021a), hair follicle cells (Gola et al., 2012), *etc.* Although the successful differentiations of MSCs into nerve cells, sweat gland cells and hair follicle cells have not only verified the multiple differentiation potential of MSCs, few studies have explored the possibility of directional differentiation of MSCs towards LESCs under specific biochemical and physical cues. Here, we explored the differentiation of MSCs towards LESCs by culturing the sodium alginate-gelatin 3D bio-printed matrix loaded with MSC in medium containing inducing biochemical cues. We hypothesized that adult MSCs with multi-lineage and trans-dermal differentiation potencies can differentiate towards LESCs under specific inducing conditions.

## 2 Methods and materials

### 2.1 Isolation and culturing of mouse mesenchymal stem cells (mMSCs)

The extraction of mMSCs was referred to previous studies (Liu et al., 2020; Liu et al., 2021a). One-week-old C57BL/6 mice were sacrificed by cervical dislocation and then soaked in 75% alcohol (Wanchun, Beijing) for 10 min to be thoroughly disinfected. One-week-old C57BL/6 mice were purchased from the SPF Laboratory Animal Center (Beijing, China). The sacrificed and sterilized mice were placed in 100 mm cell culture dishes (Corning, United States). After the skin, fascia, and muscle of mouse legs were carefully peeled off, femurs and tibiae were dissected and placed in 100 mm cell culture dishes (Corning, United States). Femurs and tibias were processed into pieces of approximately 1 mm^3^ using hemostats and ophthalmic scissors. The medium was discarded and a freshly prepared collagenase type I solution [0.25% collagenase type I, 20% fetal bovine serum, phosphate buffered saline (PBS)] was added. Culture dishes (Corning, United States) holding bone fragments were placed at 37°C for 45–60 min with shaking every 5 min. After being shaken the last time, primary mMSCs were harvested after centrifugation at 300–400 g for 10 min and incubated with complete MesenCult™ Medium (mouse) at 37°C, 5% CO_2_. After 72 h the medium was changed and cells were passaged at 80% confluence in 0.05% trypsin in 0.02% ethylenediaminetetraacetic acid (EDTA, Gibco, Canada). The whole process was completed under sterile conditions. All animal experiments were performed in accordance with the guidelines of the Institutional Animal Care and Use Committee of Chinese People’s Liberation Army (PLA) General Hospital (Beijing, China). All experimental protocols were approved by the Institutional Animal Care and Use Committee of Chinese PLA General Hospital (Beijing, China).

### 2.2 Preparation, mechanical property and microscopic detection of sodium alginate-gelatin composite matrices

1 g of sodium alginate (180,947–100G, Sigma, United States) and 3 g of gelatin (G9382-100G, Sigma, United States) were weighed using an electronic scale (JM-B2003, China). The weighed sodium alginate and gelatin were mixed evenly and then transferred to a volumetric flask filled with 100 mL of ultrapure water. The sodium alginate-gelatin blends were stirred until homogeneously mixed. The sodium alginate-gelatin blends were sterilized by pasteurization (70°C, 30 min; 4 °C, 5 min; repeated 3 times). The sodium alginate-gelatin blends were sealed and stored at 4°C for subsequent experiments.

Compression tests and tensile tests were used to measure the Young’s modulus of sodium alginate-gelatin composite matrices with encapsulated cells (Xu et al., 2019; Yao et al., 2019; Liu et al., 2021b). The cylinder samples were made into height (h) of 16 mm and diameter (d) of 16 mm for compression tests, and then these cylinder samples were cross-linked in a 2.5% CaCl_2_ for 24–48 h. The rectangular samples [lengths (L) were 30 mm, widths (w) were between 2 mm and 5 mm, and heights (h) were between 5 mm and 10 mm) were used for tensile tests and then these rectangular samples were cross-linked in a 2.5% CaCl_2_ for 24–48 h. A universal tensile machine device (INSTRON, Model 5,567) was used to perform the compression tests (a load of 100 N and a compression speed of 3 mm/min) and the tensile tests (a load of 100 N and a tensile speed of 50 mm/min) at room temperature. The measurements for each group were repeated 3 times and averaged. The Young’s modulus (E) was calculated as a slope of the initial linear region (0%–10% of strain) of the stress-strain curve Equation 1 (Xu et al., 2019; Liu et al., 2021a):

E=σ/ε (1)

where σ is the stress, ε is the strain.

The microstructure of the sodium alginate-gelatin blends was analyzed using a scanning electron microscope (SEM S-4800, HITACHI, Tokyo, Japan). Non-destructive cross-sections were taken of samples after being freeze-dried for 48 h (Christ Alpha 2-4 LD freeze-dryer) and then sputter-coated with gold (20 nm, Edwards Sputter Coater).

### 2.3 Fabrication of MSC-loaded 3D bio-printed matrices

MSCs suspension (1 mL, 1.0 × 10^7^ cells) and 9 mL sodium alginate-gelatin blends were mixed well and sealed in sterile printed syringes. The sodium alginate-gelatin blends mixed with MSC suspension in the syringe was printed into a 60 mm cell culture dish by manual extrusion to construct a 3D bio-printed matrix loaded with MSCs. The bio-printed matrices were cross-linked in sterile 2.5% calcium chloride (CaCl_2_, CC3061-500G, Coolaber) for 10 min and placed in a sterile incubator at 37°C and 5% CO_2_. Previous studies have shown that the directional differentiation of MSCs was inseparable from the corresponding induction conditions, and the differentiation of stem cells into LESCs was inseparable from the orderly addition of specific induction factors. Therefore, the first group named the control group was cultured with DMEM medium (31,600-500 mL, solaibao, China) containing 10% fetal bovine serum (DMEM-FBS) and the second group named the induced group was cultured with DMEM medium containing 10% fetal bovine serum, N2 (17,502–048, Gibco, United States), B27 (17,504–044, Gibco, United States), MNA (11,140,050, Gibco, United States) and specific cell-inducing factors (N2/B27-DMEM-FBS). The mediums were changed once a day during the 7-day incubation period. The order of adding specific cell-inducing factors was as follows: *1*) from day 0 to day 1, the induced groups were cultured in N2/B27-DMEM-FBS medium containing 100 ng/mL Noggin (PHC1506, Gibco, United States), 20 ng/mL BMP4 (PHC9534, Gibco, United States), 20 ng/mL BMP7 (PHC7204, Gibco, United States) and 100 ng/mL FGF-basic (FGF-2/bFGF, PHG0266, Gibco, United States); *2*) from day 1 to day 3, the induced groups were cultured in N2/B27-DMEM-FBS medium containing 100 ng/mL Noggin, 20 ng/mL BMP4, 20 ng/mL BMP7, 100 ng/mL FGF-basic (FGF-2/bFGF) and 20 ng/mL Wnt-3a (315–20-10, PeproTech, United States); *3)* from day 3 to day 7, the induced groups were cultured in N2/B27-DMEM-FBS medium containing 100 ng/mL FGF-basic (FGF-2/bFGF) and 20 ng/mL Wnt-3a.

### 2.4 Cell viability detection in 3D bio-printed matrix

The LIVE/DEAD^®^ Viability/Cytotoxicity Kit (Invitrogen, United States) was used to stain the 3D bio-printed matrices loaded with MSCs for analysis of the viability of cells in the matrices at day 1, 3, 5 and 7 after printing. Fluorescence images were collected with an inverted fluorescence microscope (Leica, BMI4000, Germany).

### 2.5 Immunofluorescence analysis

The matrices were fixed by soaking in 4% formaldehyde solution for 20 min. After the formaldehyde solution was discarded, the fixed matrices were soaked in lysate solution (Ouyang et al., 2015) (0.06 M sodium citrate dihydrate, 0.15 M sodium chloride and 0.02 M EDTA in deionized water) and then placed on a horizontal shaker for 10 min. After the matrices were completely lysed, the cells were collected by low-speed centrifugation at 400 *g*. Cells were resuspended in PBS, transferred to adhesive slides, and dried at 60°C for 15 min. Anti-PAX6 rabbit recombinant monoclonal antibody (1:200, ab195045, Abcam), Anti-alpha A Crystallin/CRYAA rabbit recombinant monoclonal antibody (1:200, ab181866, Abcam) and Anti-Alpha B Crystallin rabbit recombinant monoclonal antibody (1:200, ab76467, Abcam) were prepared respectively with primary antibody solution [0.3% Triton X-100 (Sigma-Aldrich, United States); 5% goat serum (Zsbio, China); PBS]. Slides were incubated with prepared Anti-alpha A Crystallin/CRYAA rabbit recombinant monoclonal antibody (1:200, ab181866, Abcam) and Anti-Alpha B Crystallin rabbit recombinant monoclonal antibody (1:200, ab76467, Abcam) at 4°C for 12–16 h. And then, after washing 3 times with PBS, the slides were incubated in conjugated AffiniPure goat anti-rabbit (1:200, CoraLite594, SA00013-4) for 2 h at room temperature and protected from light. Nuclei were counterstained with DAPI Fluoromount-G (0100–20, Southern Biotech) and coverslips were mounted on the slides, and fluorescence images were collected using a laser scanning confocal microscope (Leica, SP8 FALCON, Germany).

### 2.6 Real-time quantitative polymerase chain reaction (RT-qPCR) analysis to detect the differentiation of MSCs towards LESCs in the matrices

Cells obtained after decomposition of the matrices were completely lysed in Trizol (Invitrogen), and then transferred to 1.5 mL eppendorf (EP) tubes, 1 mL per tube. The extraction of total RNA was carried out according to the aforementioned method (Wang et al., 2020; Yao et al., 2020), and the PrimeScript™ RT kit (TaKaRa, China) was used for reverse transcription. TB Green™ Premix Ex Taq™ II (TaKaRa, China) was used to amplify cDNA. The PCR process was performed using QuantStudio 5 (Thermo-Fisher, United States). All data were analyzed using the C(t) value comparison method. The primers used were listed in Table 1.

**TABLE 1:** Primer sequences.

Groups	Primer	Sequences	
Genes associated with lens epithelial stem cell phenotypes	*Bfsp1*	Forward	CTC​CTC​AAG​TGT​CCC​TGG​TTA
		Reverse	GAT​CTG​CTC​GTT​GTA​AAG​CTG​TA
	*Cryaa*	Forward	CAG​CAT​CCT​TGG​TTC​AAG​CG
		Reverse	GGC​GGT​AGT​AGG​GGC​TGA​T
	*Cryab*	Forward	GTT​CTT​CGG​AGA​GCA​CCT​GTT
		Reverse	GAG​AGT​CCG​GTG​TCA​ATC​CAG
	*Pax6*	Forward	TAC​CAG​TGT​CTA​CCA​GCC​AAT
		Reverse	TGC​ACG​AGT​ATG​AGG​AGG​TCT
	*Mip*	Forward	CTG​TCC​GAG​GAA​ACC​TAG​CG
		Reverse	TCG​TCG​TAT​GTA​GCA​AAG​ATG​C
Genes related to Crystalline lens development signaling pathways	*β-catenin*	Forward	ATG​GAG​CCG​GAC​AGA​AAA​GC
		Reverse	CTT​GCC​ACT​CAG​GGA​AGG​A
	*Jag1*	Forward	CCT​CGG​GTC​AGT​TTG​AGC​TG
		Reverse	CCT​TGA​GGC​ACA​CTT​TGA​AGT​A
	*Notch3*	Forward	TGC​CAG​AGT​TCA​GTG​GTG​G
		Reverse	CAC​AGG​CAA​ATC​GGC​CAT​C
	*Fgfr2*	Forward	AAT​CTC​CCA​ACC​AGA​AGC​GTA
		Reverse	CTC​CCC​AAT​AAG​CAC​TGT​CCT
	*Fgfr3*	Forward	TGG​ATC​AGT​GAG​AAT​GTG​GAG​G
		Reverse	CCT​ATG​AAA​TTG​GTG​GCT​CGA​C
	*Bmp4*	Forward	TTC​CTG​GTA​ACC​GAA​TGC​TGA
		Reverse	CCT​GAA​TCT​CGG​CGA​CTT​TTT
	*Bmp7*	Forward	ACG​GAC​AGG​GCT​TCT​CCT​AC
		Reverse	ATG​GTG​GTA​TCG​AGG​GTG​GAA
	*Tgf-β1*	Forward	CTC​CCG​TGG​CTT​CTA​GTG​C
		Reverse	GCC​TTA​GTT​TGG​ACA​GGA​TCT​G
	*Tgf-β2*	Forward	TCG​ACA​TGG​ATC​AGT​TTA​TGC​G
		Reverse	CCC​TGG​TAC​TGT​TGT​AGA​TGG​A

### 2.7 Statistical analysis

The data shown were expressed as means ± standard deviations. International business machine supplementary power supply set (IBM SPSS) software 29 was used to perform the statistical analysis. Two-tailed Student’s t-test was used to analyze the differences between two groups and one-way ANOVA *post hoc* test was used to analyze the differences between multiple groups. The *p*-value less than 0.05 were considered statistically significant.

## 3 Results

### 3.1 Demonstration of physical properties of the sodium alginate-gelatin composite hydrogel

**FIGURE 1:**
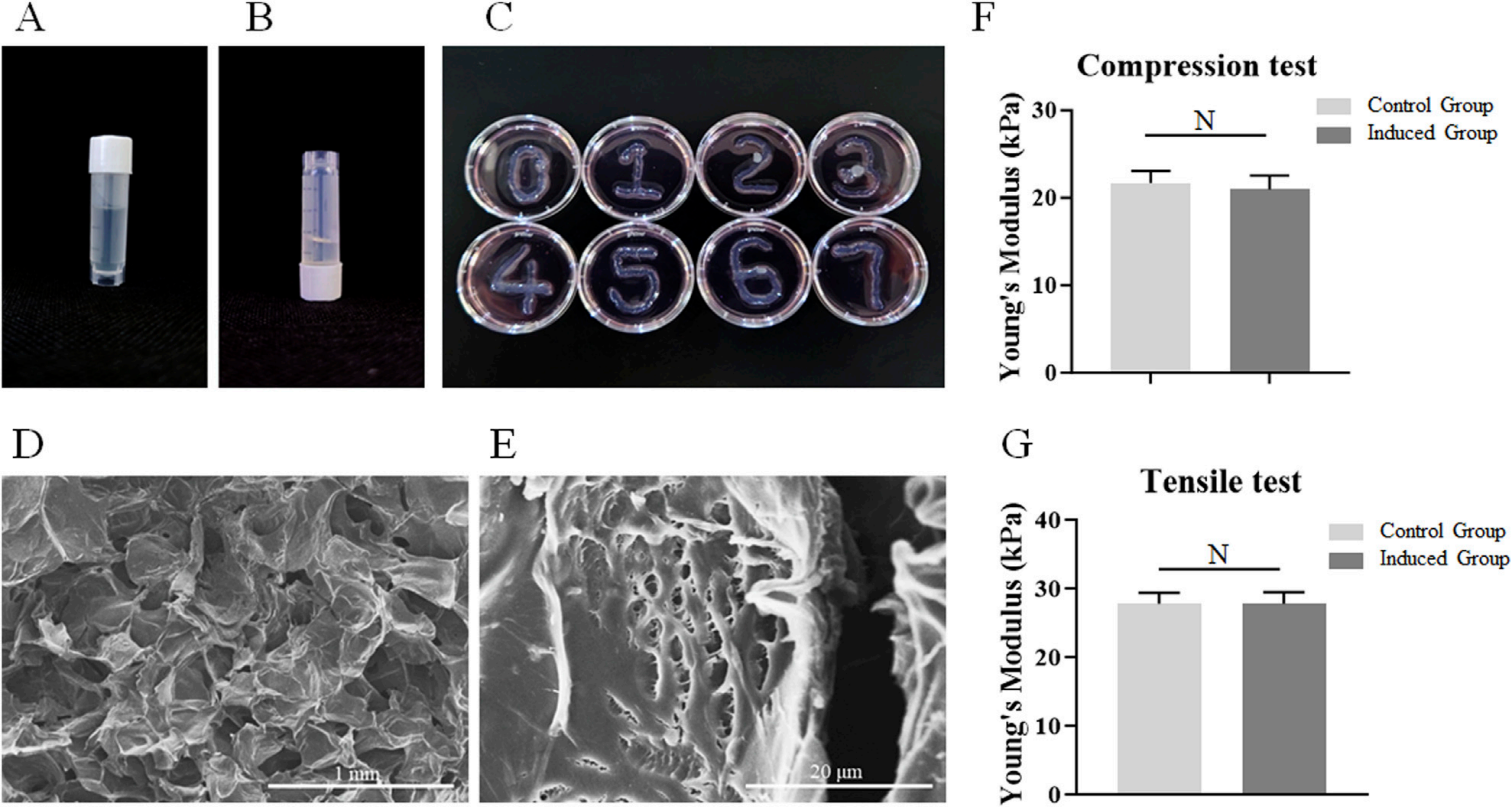
Demonstration of physical properties of the sodium alginate-gelatin composite hydrogel. **(A)** The gelatinous state of the matrices at 4°C. **(B)** The dissolved state of the matrices at room temperature. **(C)** The morphology of the matrices during the 7-day culture period. **(D–E)**: The microstructures of matrix observed using SEM (**(D)** scale bar 1 mm; **(E)** scale bar 20 μm). **(F–J)**: The Young’s modulus of the control group and the induced group, Note: N = not significant.

At room temperature, the sodium alginate-gelatin composite hydrogel presented a liquid state with strong fluidity during the upside-down process of the frozen storage tube (Figure 1B). At 4°C, the sodium alginate-gelatin composite hydrogel showed a transparent gel state (Figure 1A). After being cross-linked with 2.5% CaCl_2_, the matrices were placed in the mediums and incubated at 37°C for 7 days. The morphology of the matrices did not change significantly during the 7 days culture period, and the matrices could continue to maintain a 3D structures (Figure 1C). SEM images showed that the microstructure of sodium alginate-gelatin composite hydrogel was a porous structure from low resolution to high resolution (Figures 1D,E; Supplementary Figure S1). There was no significant difference in Young’s modulus between the control group (21.68 ± 1.38 kPa) and the induced group (20.94 ± 1.59 kPa) in compression tests. There was no significant difference in Young’s modulus between the control group (27.78 ± 1.56 kPa) and the induced group (27.74 ± 1.70 kPa) in tensile tests (Figures 1F,G).

### 3.2 Fabrication of MSC-loaded 3D bio-printed matrices

**FIGURE 2:**
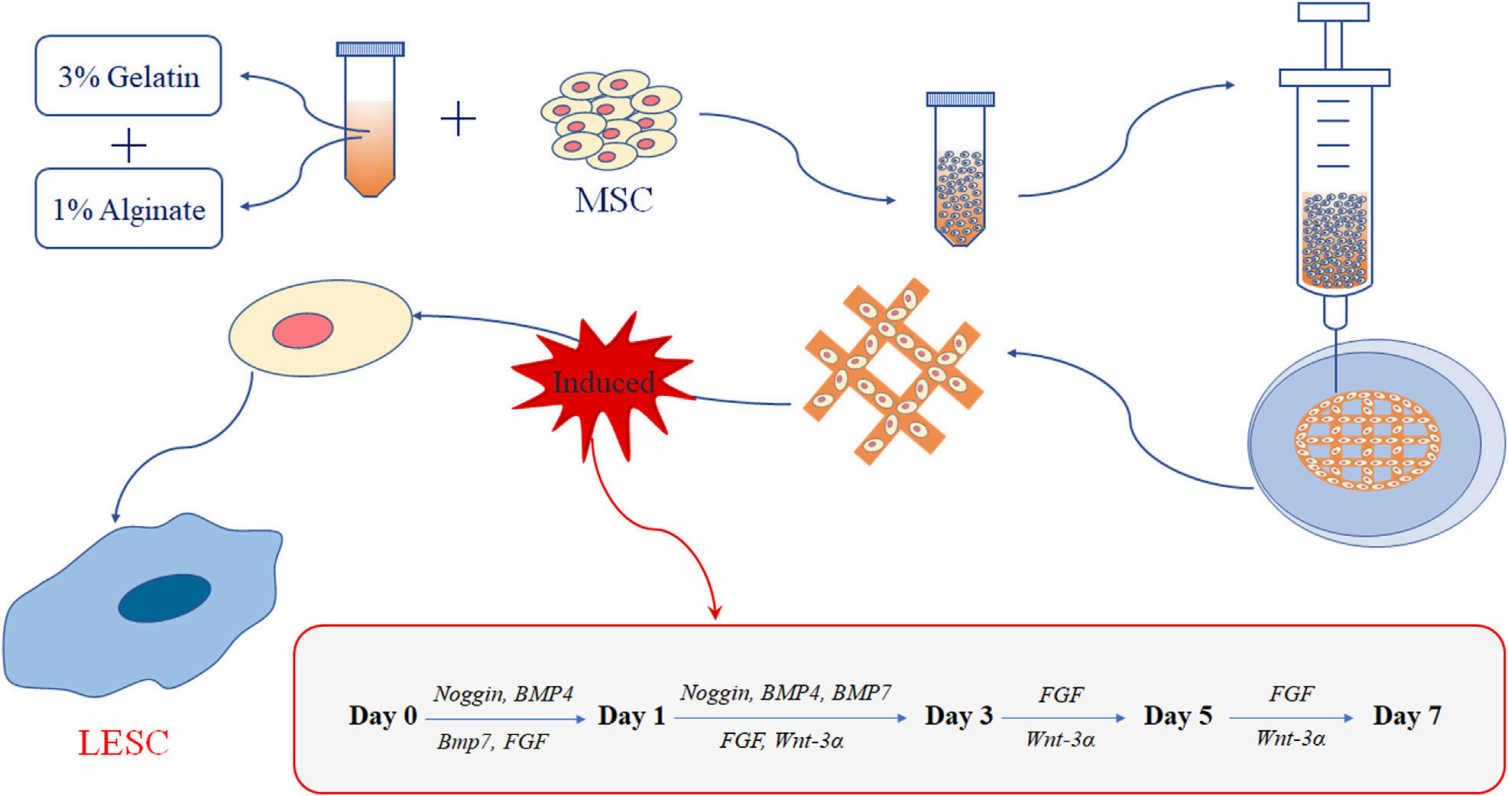
MSCs encapsulated in the 3D bio-printed matrices.

Based on the results of previous studies, we dissolved a mixture of sodium alginate and gelatin in ultrapure water to form a composite hydrogel with a final concentration of 1% sodium alginate and 3% gelatin (Figure 2). We used extrusion printing to print the gel-state composite hydrogel into a three-dimensional structure, and then cross-linked it with 2.5% CaCl_2_. We divide the cross-linked matrices into two groups. The first group was cultured in DMEM-FBS for 7 days, and was recorded as the control group. The second group was cultured in N2/B27-DMEM-FBS for 7 days, and was recorded as the induced group.

### 3.3 Cell viability of loaded MSCs in 3D bio-printed matrices

**FIGURE 3:**
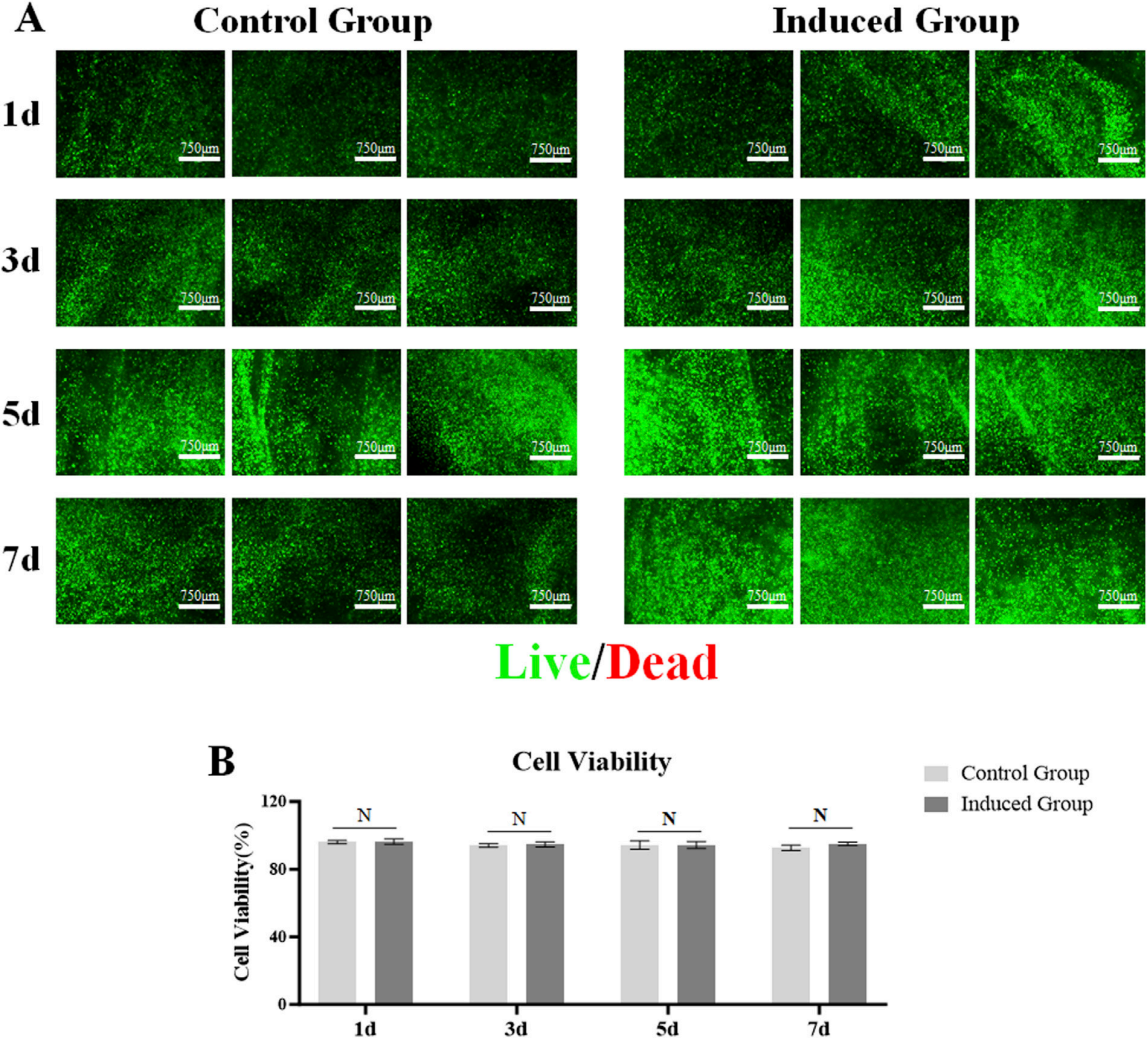
Viability of MSCs encapsulated in the 3D bio-printed matrices. **(A)** Live cells were stained with green fluorescence calcein-acrylamide and dead cells were stained with red fluorescence ethidium Dimer-1 (scale bar 750 μm); **(B)** A histogram of Cell Viability of MSCs encapsulated in the two groups, Note: N = not significant.

Good biocompatibility, that is, cell viability, is a crucial factor in constructing 3D bio-printing matrices loaded with cells, and is the basis for the MSCs loaded in the matrices to be further induced to differentiate towards LESCs. Therefore, during the 7-day culture period, the viability statuses of the MSCs loaded in the matrices of the control group and the induced group were detected on days 1, 3, 5, and 7 respectively. Cell live-death staining was used to detect the viability statuses of MSCs loaded in the matrices. We found that MSCs loaded in the matrices of the control group and the induced group were able to be maintained high cell viability throughout the 7-day culture period (Figure 3A). There was no significant difference in cell activity between the control group and the induced group (Figure 3B).

### 3.4 Changes in genes associated with lens epithelial stem cell phenotypes

**FIGURE 4:**
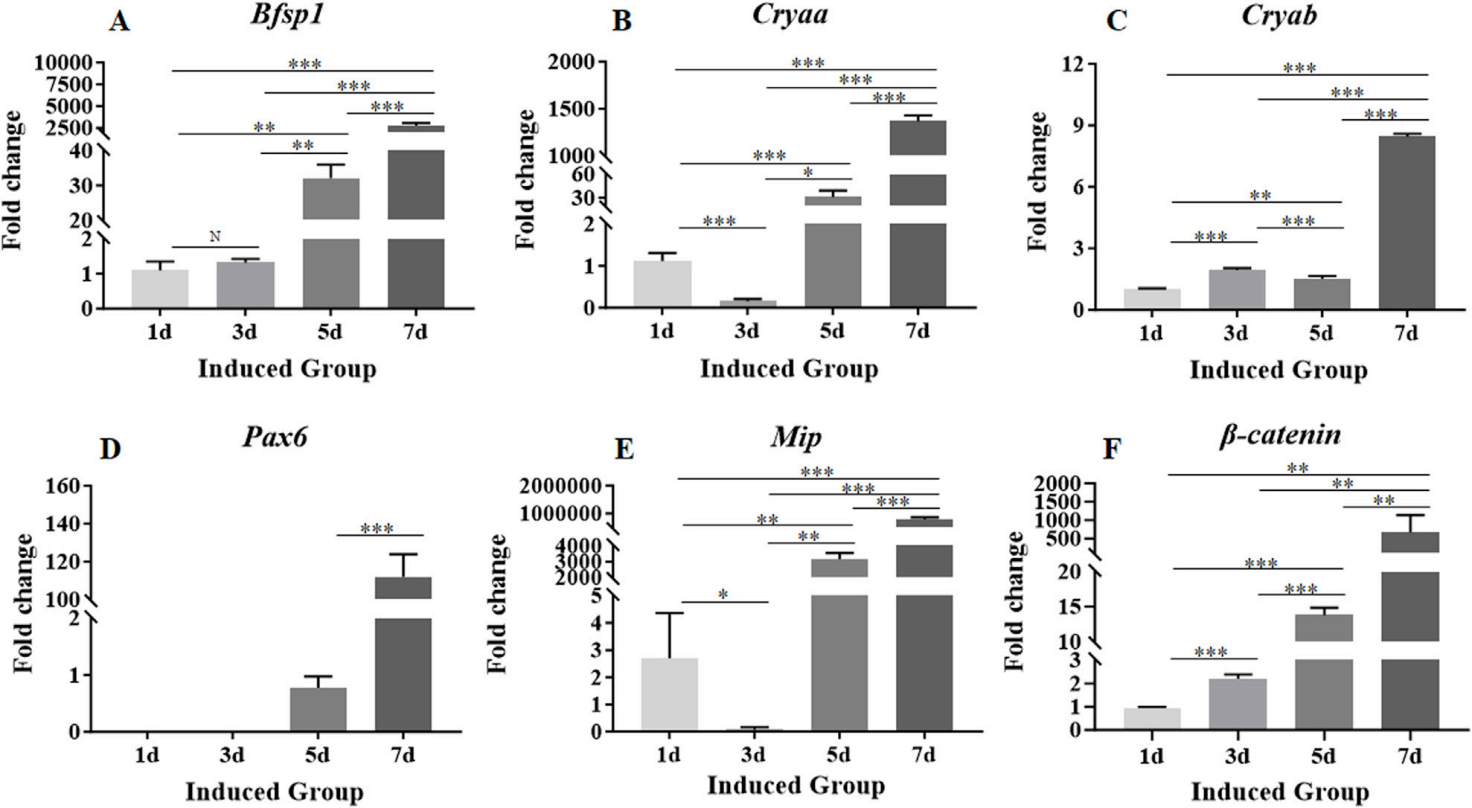
Expression levels of genes associated with the phenotypes of LESCs and signaling pathway involved in crystalline lens development in MSCs encapsulated in the 3D bio-printed matrices of the induced group. **(A–F)**: The expression levels of *Bsfp1*, *Cryaa*, *Cryab*, *Pax6*, *Mip* and *β-catenin*. Gene expression was normalized relative to *Gapdh* expression. Note: N = not significant, *p < 0.05, **p < 0.01, ***p < 0.001.

In order to detect whether the MSCs loaded in the 3D bio-printed matrices could be induced to differentiate towards LESCs in 7-day culture period, the expression levels of genes related to the phenotypes of LESCs in the induced group were detected on day 1, day 3, day 5, and day 7. PCR results showed that the gene expressions associated with phenotypes of LESCs were not detected throughout the 7-day culture period in the control group (Supplementary Figure S2). Therefore, only the experimental results of the induced group were shown in the following presentation of the research results. On day 1, the expression levels of *Bsfp1*, *Cryaa*, *Cryab*, and *Mip* were detected in the induced group (Figures 4A–C,E). On day 3, the expression level of *Bsfp1* was slightly increased compared with that on day 1, but there was no significant difference between the two time points (Figure 4A). From day 5 to day 7, the expression level of *Bsfp1* was continuously increased in the induced group, and the expression levels of *Bsfp1* on day 5 and day 7 were significantly different from the expression level of *Bsfp1* on day 3 (Figure 4A). On day 3, the expression levels of *Cryaa* and *Mip* were significantly decreased compared with those on day 1 (Figures 4B,E). The expression levels of *Cryaa* and *Mip* were significantly increased from day 5 to day 7 (Figures 4B,E). The expression level of *Cryab* was fluctuated greatly over the 7-day culture period (Figure 4C). Although the expression level of *Cryab* was increased slightly on day 3 and decreased slightly on day 5, the increased of *Cryab* expression on day 3 was significantly lower than that on day 1 and the decreased of *Cryab* expression on day 5 was also significantly higher than that on day 3 (Figure 4C). On day 7, the expression level of *Cryab* was significantly increased compared with that on day 1, 3 and 5 (Figure 4C). The expression level of *Pax6* in the induced group was first detected on day 5 (Figure 4D). The expression level of *Pax6* was increased from day 5 to day 7 (Figure 4D). The expression level of *Pax6* on day 7 was significantly higher than that on day 5 (Figure 4D).

### 3.5 Changes in genes associated with pathways involved in crystalline lens development

**FIGURE 5:**
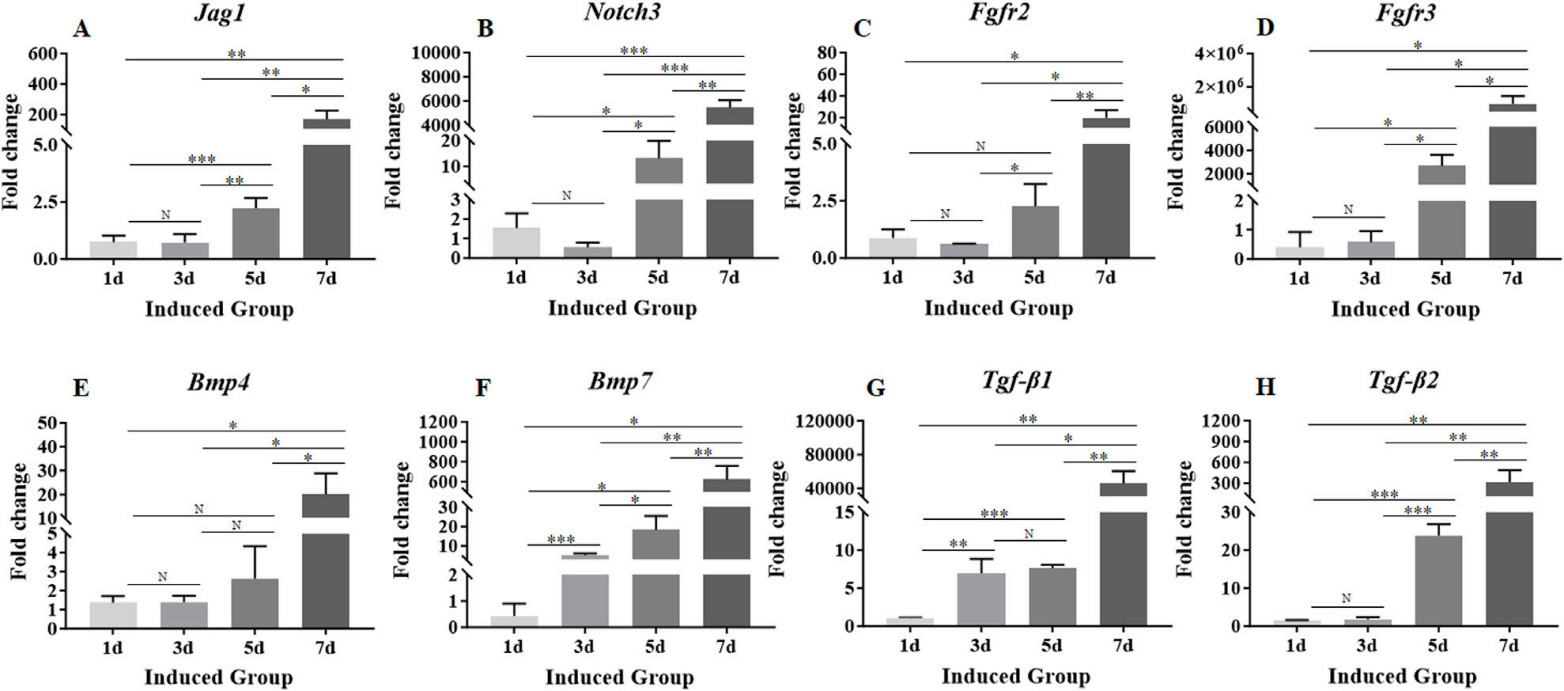
Expression levels of genes associated with signaling pathways involved in crystalline lens development. **(A–H)**: The expression levels of *Jag1*, *Notch3*, *Fgfr2*, *Fgfr3*, *Bmp4*, *Bmp7*, *Tgf-β1* and *Tgf-β2*. Gene expression was normalized relative to *Gapdh* expression. Note: N = not significant, *p < 0.05, **p < 0.01, ***p < 0.001.

Expression levels of *β-catenin*, *Jag1*, *Notch3*, *Bmp4*, *Bmp7*, *Fgfr2*, *Fgfr3*, *Tgf-β1* and *Tgf-β2* genes associated with signaling pathways involved in crystalline lens development were detectable on day 1 (Figures 4F, 5). The expression level of *β-catenin* was consistently and significantly increased at each time point throughout the 7-day culture period (Figure 4F). The expression levels of other genes were fluctuated during the whole 7-day culture period (Figure 5). The expression level of *Jag1* on day 3 was not significantly different from that on day 1 (Figure 5A). The expression level of *Jag1* was significantly increased from day 5 to day 7 (Figure 5A). The expression level of *Notch3* on day 3 was slightly lower than that on day 1, but the difference was not statistically significant (Figure 5B). From day 5 to day 7, the expression level of *Notch3* was continuously increased with significant difference (Figure 5B). From day 1 to day 5, the expression level of *Bmp4* was showed a slight increasing trend but no statistical difference (Figure 5E). The expression level of *Bmp4* was significantly increased on day 7 compared with that on day 1, day 3 and day 5 (Figure 5E). The expression level of *Bmp7* was significantly increased from that on day 1 to day 7 (Figure 5F). On day 3, the expression level of *Fgfr2* was slightly decreased but not significantly different from that on day 1 (Figure 5C). On day 5, the expression level of *Fgfr2* was significantly increased compared with that on day 3 (Figure 5C). However, there was no significant difference in the expression level of *Fgfr2* between that on day 5 and day 1 (Figure 5C). On day 7, the expression level of *Fgfr2* was significantly increased compared with that on day 5, day 3, and day 1 (Figure 5C). There was no significant difference in the expression of *Fgfr3* between that on day 3 and day 1 (Figure 5D). The expression level of *Fgfr3* was significantly increased from day 5 to day 7 (Figure 5D). On day 3, the expression level of *Tgf-β1* was significantly increased compared with that on day 1 (Figure 5G). There was no significant change in the expression level of *Tgf-β1* on day 5 compared with that on day 3 (Figure 5G). On day 7, the expression level of *Tgf-β1* was significantly increased compared with that on day 5, day 3 and day 1 (Figure 5G). On day 3, the expression level of *Tgf-β2* did not change significantly compared with that on day 1 (Figure 5H). The expression level of *Tgf-β2* was significantly increased from day 5 to day 7 (Figure 5H).

### 3.6 Differentiation of MSCs loaded in 3D bio-printed matrices towards LESCs

**FIGURE 6:**
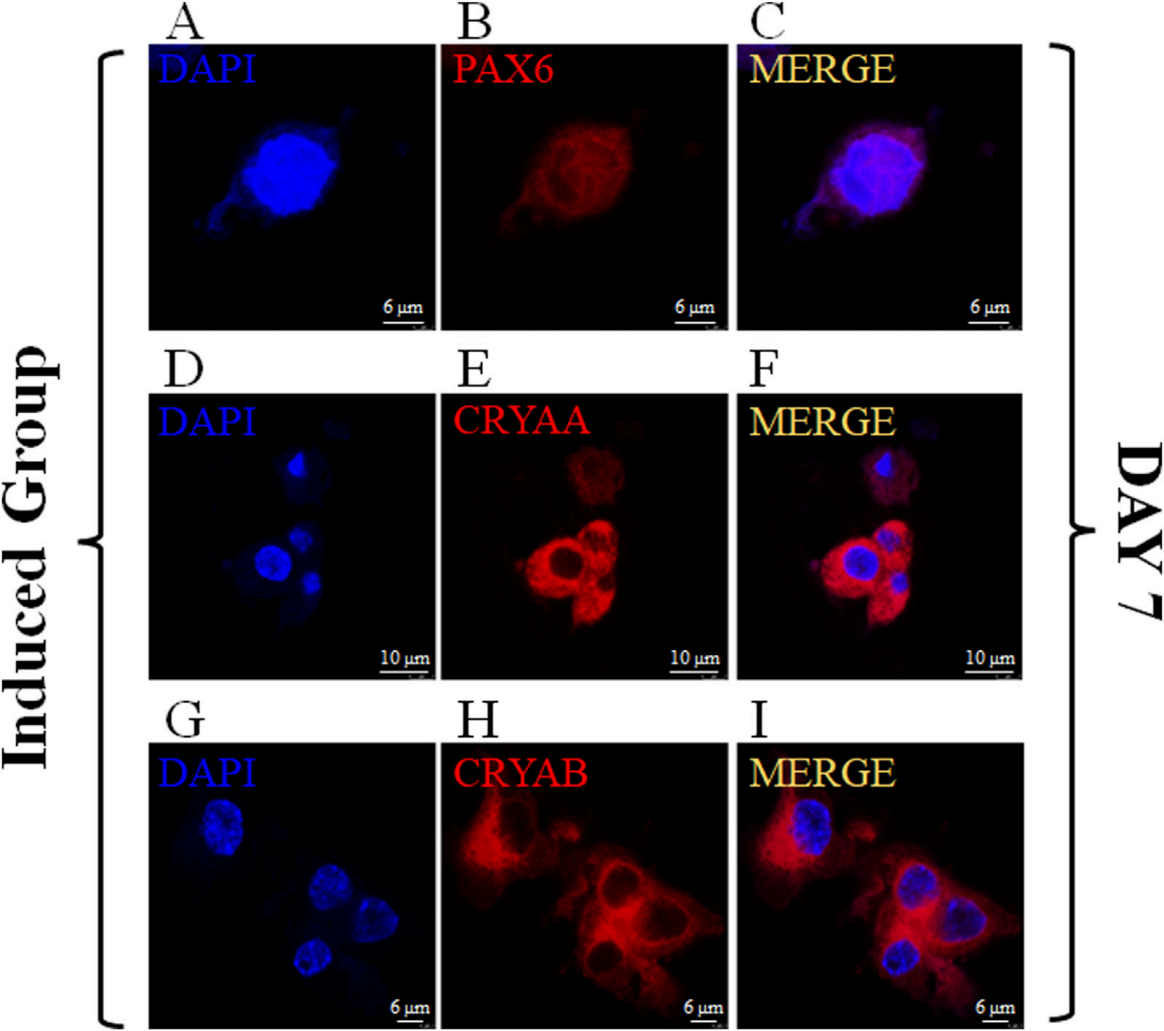
Expression of PAX6、CRYAA and CRYAB on MSCs encapsulated in the induced group on Day 7. The immunofluorescence staining of PAX6、CRYAA and CRYAB, Cell nuclei were stained with DAPI (**(A–C)**: scale bar 6 μm; **(D–F)**: scale bar 10 μm; **(G–I)**: scale bar 6 μm).

PAX6, CRYAA and CRYAB are commonly used markers to detect LESCs in previous studies (Yang et al., 2010; Lin et al., 2016). Immunofluorescence staining was used to detect the expression of PAX6, CRYAA and CRYAB in control group and induced group to determine whether the loaded MSCs could be differentiated towards LESCs after induction (Figure 6; Supplementary Figure S3). Immunofluorescence staining showed that transcription factor PAX6 was strongly expressed in the nucleus on day 7 (Figure 6). Immunofluorescence staining images of CRYAA and CRYAB also showed that they were clearly expressed at day 7 (Figure 6).

## 4 Discussion

The uncertainty of the treatment of traumatic cataract mainly lies in the complexity and difficulty of the surgical method, the large damage to the ocular tissue during the perioperative period, the unstable recovery of postoperative vision, and the many complications. Therefore, the exploration of autogenous rapid lens regeneration method has become an innovative concept for the treatment of traumatic cataract. The three-stage induction system has been proven to be able to effectively induce the differentiation of hESCs into lens precursor cells and lens-like bodies, and the important role of endogenous LESCs in lens regeneration has also been confirmed (Yang et al., 2010; Lin et al., 2016). Due to the ethical controversy of hESCs and the difficulty in obtaining endogenous LESCs, we intended to explore the possibility of differentiation of MSCs, which are recognized to have multi-lineage differentiation potential, towards LESCs by three-stage induction system. We have previously demonstrated that 3D bio-printed matrices using sodium alginate-gelatin composite hydrogel as bioink could induce the MSCs loaded in the hydrogels to rapid differentiate into cells derived from different germ layers such as bone cells, adipocytes, nerve cells, and sweat gland cells by the conditions of specific biochemical cues (Chen et al., 2016; Jin et al., 2019; Pajarinen et al., 2019; Liu et al., 2020; Liu et al., 2021b). Moreover, our previous studies have also confirmed that the biophysical cues, such as stiffness, brought by the hydrogel structures could accelerate the differentiation of MSCS into specific cells through the activation of YAP signaling pathway (Liu et al., 2020; Liu et al., 2021a). On the other hand, multiple studies have also confirmed that the biophysical cues provided by 3D bioprinting matrices using sodium alginate-gelatin composite hydrogel as bioink for loaded stem cells could promote the proliferation and differentiation of stem cells (Song et al., 2014; Liu et al., 2021b; Wang et al., 2022; Ju et al., 2023). Based on the above research results, we hypothesized that the biophysical cues provided by the MSC-loaded sodium alginate-gelatin 3D bio-printed matrices combined with the biochemical cues provided by the three-stage induction system can promote the differentiation of MSCs towards LESCs. To confirm this hypothesis, we used a composite hydrogel with a final concentration of 1% sodium alginate and 3% gelatin as the bioink. The composite hydrogel of 1% sodium alginate-3% gelatin have been proven to maintain good cell viability of MSCs and promote MSC differentiation (Liu et al., 2020; Wang et al., 2020; Yao et al., 2020; Liu et al., 2021a). The results of cell viability test showed that both the control group and the induced group in our study could maintain high cell viability during the 7-day culture period. The absence of statistical difference between the control group and the induced group indicated that the inducing factors added in the induced group in this study had no effect on cell viability. Multiple studies have shown that the porous structure of bioinks that can be used for bio-printing is beneficial to promoting cell adhesion, activity, proliferation and differentiation (Korytkin et al., 2021; Li et al., 2021; Parisi et al., 2021; Hernandez and Woodrow, 2022). The SEM results in this study showed that the microstructure of the composite hydrogel of 1% sodium alginate-3% gelatin at different magnifications was a porous-like structure. This indicated that the 1% sodium alginate-3% gelatin composite hydrogel used in this study could provide beneficial structural support for cell survival, and also provided a basis of biophysical cues for further research on the directional differentiation of MSCs. In order to exclude the effect of the Young’s modulus, that is stiffness, on the progression of MSC differentiation, we tested the Young’s modulus of the control group and the induced group through compression experiments and tensile tests. The test results of Young’s modulus of the compression experiments and the tensile tests showed that there was no significant difference between the control group and the induced group. This suggested that the difference in the degree of differentiation of MSC towards LESC between the control group and the induced group was not due to the stiffness.

The three-stage induction system used in previous study was an induction culture method with a 35-day culture period in a culture environment (Yang et al., 2010). Multiple studies on 3D bio-printing have shown that the progress of stem cell differentiation will be accelerated under 3D biophysical cues relative to two-dimensional culture environments (Liu et al., 2020; Liu et al., 2021a). Combined with our previous research results, the purpose of this research was to explore whether MSCs could be rapidly initiated the process of differentiation towards LESCs within a 7-day culture period through the combined action of biochemical cues and biophysical cues. Therefore, a three-stage induction system with a 7-day culture period under three-dimensional conditions was used in this research work. At the beginning of this work, we found that the three-stage induction system with a 7-day culture period under 3D conditions did not have an impact on cell viability. Based on this, we further evaluated the gene expression of specific markers of LESCs during the 7-day culture period through RT-qPCR analysis in order to clarify the differentiation of MSCs loaded in the induced group towards LESCs.

PAX6, as a marker of neuroectoderm, is an important regulatory factor in the nucleus during the crystalline lens development (Jonasova and Kozmik, 2008; Frederikse and Kasinathan, 2017; Koenig and Gross, 2020; Kumar and Reilly, 2020; Chang et al., 2023). BFSP1 encodes a lens-specific intermediate filament-like protein named filensin. The encoded protein is expressed in lens fiber cells after differentiation has begun (Cvekl and Camerino, 2022; Jones et al., 2022). CRYAA and CRYAB are important paralogs of each other, and both are recognized representative markers of lens cells (Andley, 2009; Kumar et al., 2013; Rumyantseva et al., 2015; Mynampati et al., 2017). The MIP protein is a member of the water-transporting aquaporins as well as the original member of the MIP family of channel proteins and the MIP protein is expressed in the ocular lens and is required for correct lens function (Chepelinsky et al., 1991; Fan et al., 2005; Bennett et al., 2016; He et al., 2023). The results of PCR showed that there was no sustained and significant upregulation of crystal specific coding genes in MSCs loaded with the control group during the whole 7-day culture (Supplementary Figure S2). These results indicated that sodium alginate and gelatin used in the study had no inducible effect on the differentiation of MSCs towards LESCs. Therefore, next only the expression levels of crystal-specific coding genes of the MSCs loaded in the induced group were shown and discussed. Based on the previous research experiences, we hypothesized that the transient high expression of some genes (*Bsfp1, Cryaa, Cryab, Mip, Jag1, Fgfr2, Bmp4, Bmp7, Tgf-β2, Notch3* and *β-catenin*) in the first 3 days of the culture period were mainly due to the influence of biophysical cues brought by the 3D matrix. The hypothesize needs to be confirmed by further experiments. Since the focus of this study was whether the three-dimensional induction matrix constructed in this study could initiate the differentiation of MSC towards the direction of LESC, we would mainly discuss the results of the inducted group. During the 7-day culture period of the inducted group, the expression levels of crystal-specific phenotype-related marker genes including *Bfsp1*, *Cryaa*, *Cryab*, *Pax6* and *Mip* were significantly upregulated. Compared with previous study, the appearance time of crystal-specific markers was significantly earlier, indicating that the biophysical cues provided by the 3D bio-printed matrices significantly promoted the differentiation of MSCs loaded in the induced group towards LESCs. In addition, the induction effect of biochemical cues on MSCs also played an important role. For example, on the first day, the expression of *Bfsp1*, *Cryaa*, *Cryab*, and *Mip* could all be detected, which might be indicated that these genes were more sensitive to regulation by biophysical cues. The expression level of *Bfsp1* was showed no significant change on day 3, while the expression levels of *Cryaa* and *Mip* were showed a significant decrease. The possible reason for this result is that the induction effect of CRYAA and MIP to biophysical cues is fast but not durable, so the high expression of CRYAA and MIP was detected on the first day of culture. We considered that the induction effect of biochemical cues on CRYAA and MIP began to appear from day 3, so the expression of CRYAA and MIP showed a slight downward trend from day 1 to day 3 of the culture period. The expression of CRYAA and MIP showed a significant upregulation trend from day 5 to day 7 of the culture period with the continuous appearance of the biochemical cue induction effect. This result might be indicated that although biophysical cues might be able to quickly activate the expression levels of genes that were highly sensitive to biophysical cues, biophysical cues could not independently maintain high expression levels of genes for a long time. This is also similar to our previous research conclusions. From day 5 to day 7, the expression levels of *Bfsp1*, *Cryaa*, *Cryab*, and *Mip* was continued to significantly increase. This might be indicated that the induction effect of biochemical cues exceeded the influence of biophysical cues in this study system in the first 5 days of the 7-day culture period. The expression level of *Pax6* was not detected in the first 3 days, but showed a significant and sustained increase on day 5 and day 7. The changes in the expression level of *Pax6* might be caused by the fact that *Pax6* was less affected by biophysical cues and mainly relied on the induction of biochemical cues.

The directed differentiation of stem cells is inseparable from the activation of relevant signaling pathways. Similarly, crystalline lens regeneration is also inseparable from the activation of a series of signaling pathways. Multiple studies have shown that several signaling pathways such as Notch, FGF, Wnt, and BMP/TGF-β are inseparable from the development of the crystalline lens (Hayashi et al., 2008; Cvekl and Wang, 2009; Bassnett and Šikić, 2017; Li et al., 2020). Therefore, clarifying the activation status of signaling pathways during the crystalline lens regeneration is the focus of the crystalline lens regeneration research. LESCs express almost all genes involved in the canonical Wnt signaling pathway (Stump et al., 2003; Chen et al., 2004; Cain et al., 2008). The canonical Wnt signaling pathway is activated after Wnt ligand binds to the receptor complex, thereby regulating downstream *β-catenin*. A previous study found that *β-catenin* knockout in embryonic lens progenitor cells severely disrupted the development of lens structure (Carvajal-Gonzalez et al., 2016), indicating that this pathway plays an irreplaceable role in maintaining the number of lens progenitor cells during early development. The sustained and significant increase in the expression level of *β-catenin* throughout the 7-day culture period not only indicated the activation of the Wnt signaling pathway, but might be also indicated to a certain extent that *β-catenin* responded sensitively to both biochemical and biophysical cues. This result was consistent with our previous findings. The Notch signaling pathway determines cell fate through paracrine substrates. The function of the Notch signaling pathway includes maintaining the activity of stem cells and progenitor cell populations. Studies have confirmed that Notch3 is highly expressed in LESCs (Saravanamuthu et al., 2012). There are four functional Notch ligands (Dll1, Dll4, Jag1, Jag2) in mammalian cells, among which Jag1 is mainly expressed in differentiated fiber cells (Rowan et al., 2008; Dawes et al., 2014). Although the expression levels of *Jag1*, *Notch3*, *Fgfr2* and *Fgfr3* were changed slightly from the day 1 to day 3, there were no significant difference. From day 5 to day 7, the expression levels of *Jag1*, *Notch3*, *Fgfr2* and *Fgfr3* were continuously and significantly increased, which were similar to the expression level trends of some genes encoding lens-specific markers. This result was also consistent with existing research results, that was, there was a synergistic effect between the FGF signaling pathway and the Notch signaling pathway in in vitro experiments. Previous study has shown that FGF in LECs can trigger Jag1 and Notch2, and then further promote the differentiation of lens progenitor cells into lens fiber cells (Saravanamuthu et al., 2009). On the other hand, the activation of *Jag1* induced by FGF also depends on the Notch signaling pathway (Saravanamuthu et al., 2009; Aujla et al., 2013; Dawes et al., 2014). A previous study found that BMP inhibitors can block the differentiation of lens fiber cells induced by FGF (Boswell et al., 2008), which indicates that the BMP signaling pathway also plays an important role in lens development. BMP and TGF-β ligands are not only expressed in the lens, but BMP and TGF-β are essential in the formation of the lens placode (Rajagopal et al., 2009). This indicates that the BMP/TGF-β signaling pathway is indispensable in the formation of the lens placode. In one study, BMP4 knockout resulted in severe defects in lens lamina differentiation (Furuta and Hogan, 1998), while in another study, mice without BMP7 also have related defects in lens development (Luo et al., 1995). The expression levels of *Bmp4*, *Bmp7*, *Tgf-β1* and *Tgf-β2* was fluctuated to varying degrees in the early stage. This might be also indicated that biophysical cues have a rapid upregulation effect on the BMP/TGF-β signaling pathway, but could not maintain the activation state of the BMP/TGF-β signaling pathway for a long time. In the later stage of the 7-day culture period, the expression levels of *Bmp4*, *Bmp7*, *Tgf-β1* and *Tgf-β2* were continuously and significantly upregulated, which might be have once again verified that biochemical cues were necessary to maintain the activation state of signaling pathways. In other words, under a specific induction environment, biophysical cues could quickly upregulate the expression levels of genes encoding crystal-specific markers and activate crystal development-related signaling pathways, but biochemical cues were the important factors in promoting the continued differentiation of MSCs towards LESCs. To sum up, whether it is crystal-specific markers or crystal development-related signaling pathways, although they were highly sensitive to biophysical cues, the maintenance of the activation states of the signaling pathways and the continuous up-regulations of gene expression levels still required the blessing of biochemical cues.

On the premise that the up-regulations of gene expression levels have been confirmed, we next verified that the MSCs in this study were able to differentiate towards LESCs at the protein expression level. The results of immunofluorescence staining clearly showed that MSCs loaded in the induced group could clearly express PAX6, CRYAA and CRYAB after a 7-day culture period. The results of immunofluorescence staining showed that the MSC encapsulated in the control group could not express PAX6, and CRYAA and CRYAB after a 7-day culture period (Supplementary Figure S3). This showed that the induction system and 3D experimental model used in this study were clear and feasible.

## 5 Conclusion

**FIGURE 7:**
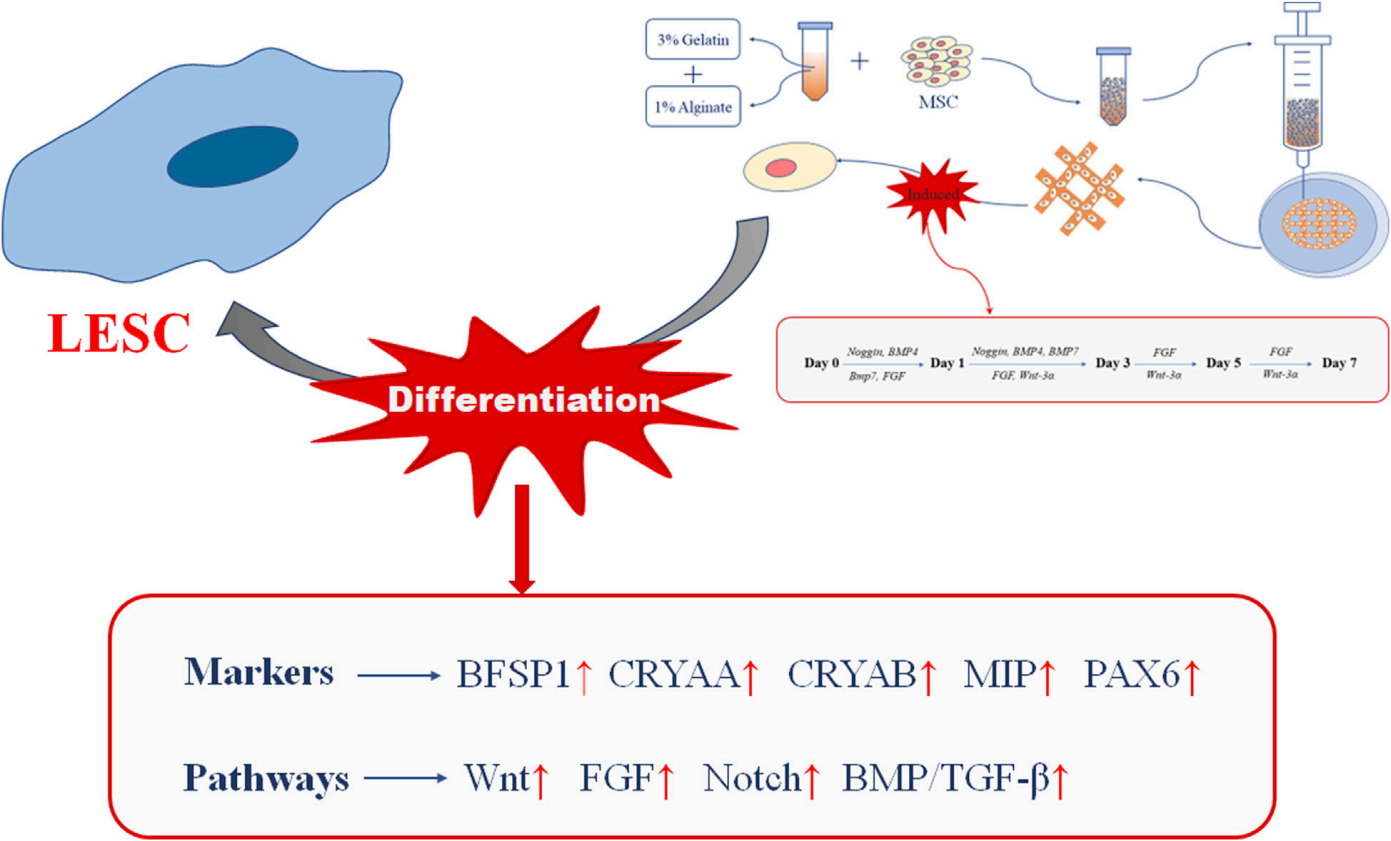
Summary of this experiment.

In summary, we studied the differentiation of MSCs towards LESCs in 3D bio-printed matrices through a modified three-stage induction system. Our results showed that MSCs encapsulated in the 3D bio-printed matrices could initiate directional differentiation towards LESCs under the combined action of biophysical cues provided by the 3D bio-printed matrices and biochemical cues provided by the three-stage induction system (Figure 7). Biophysical cues could efficiently upregulate crystal-specific markers and activate signaling pathways related to crystalline lens development, while biochemical cues could long-lastingly maintain the changes brought about by biophysical cues. Although the exact biophysical mechanisms driving these markers and signaling pathways were not yet clear, we could assume that biophysical cues played important roles in rapidly inducing lens regeneration with the support of biochemical cues. Future experiments in this study will include clarification of the relationship between biophysical cues, biochemical cues and other factors, and we expect our findings to have broad implications for rapid lens regeneration in bio-printed 3D matrices.

## Data Availability

The original contributions presented in the study are included in the article/Supplementary Material, further inquiries can be directed to the corresponding authors.

## Supplementary material

SUPPLEMENTARY FIGURE S2 Expression levels of genes associated with the phenotypes and signaling pathways involved in crystalline lens development in the control group. Gene expression was normalized relative to *Gapdh* expression. Note: N= not significant, **p* < 0.05.
