# Supplementary figures and images for: Differentiation of mesenchymal stem cells towards lens epithelial stem cells based on three-dimensional bio-printed matrix
Source: Front Cell Dev Biol. 2025 Jan 6;12:1526943. doi: 10.3389/fcell.2024.1526943 (PMC11743933; doi:10.3389/fcell.2024.1526943)

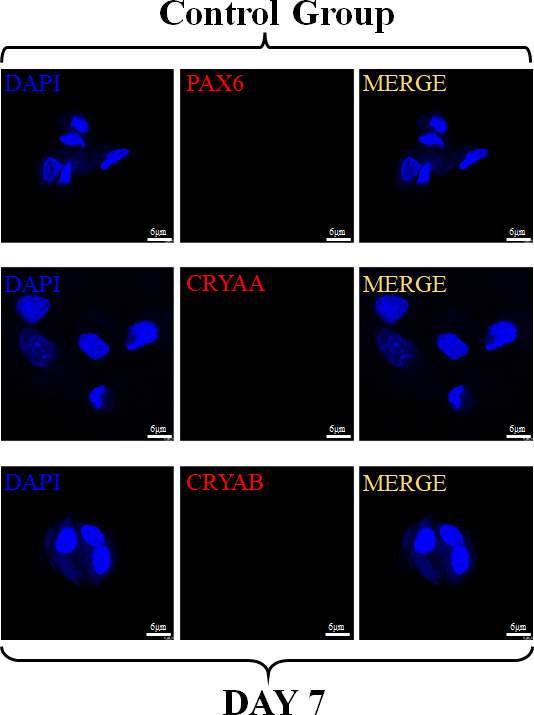

Supplement: Supplementary file 1 [file Image3.tif]

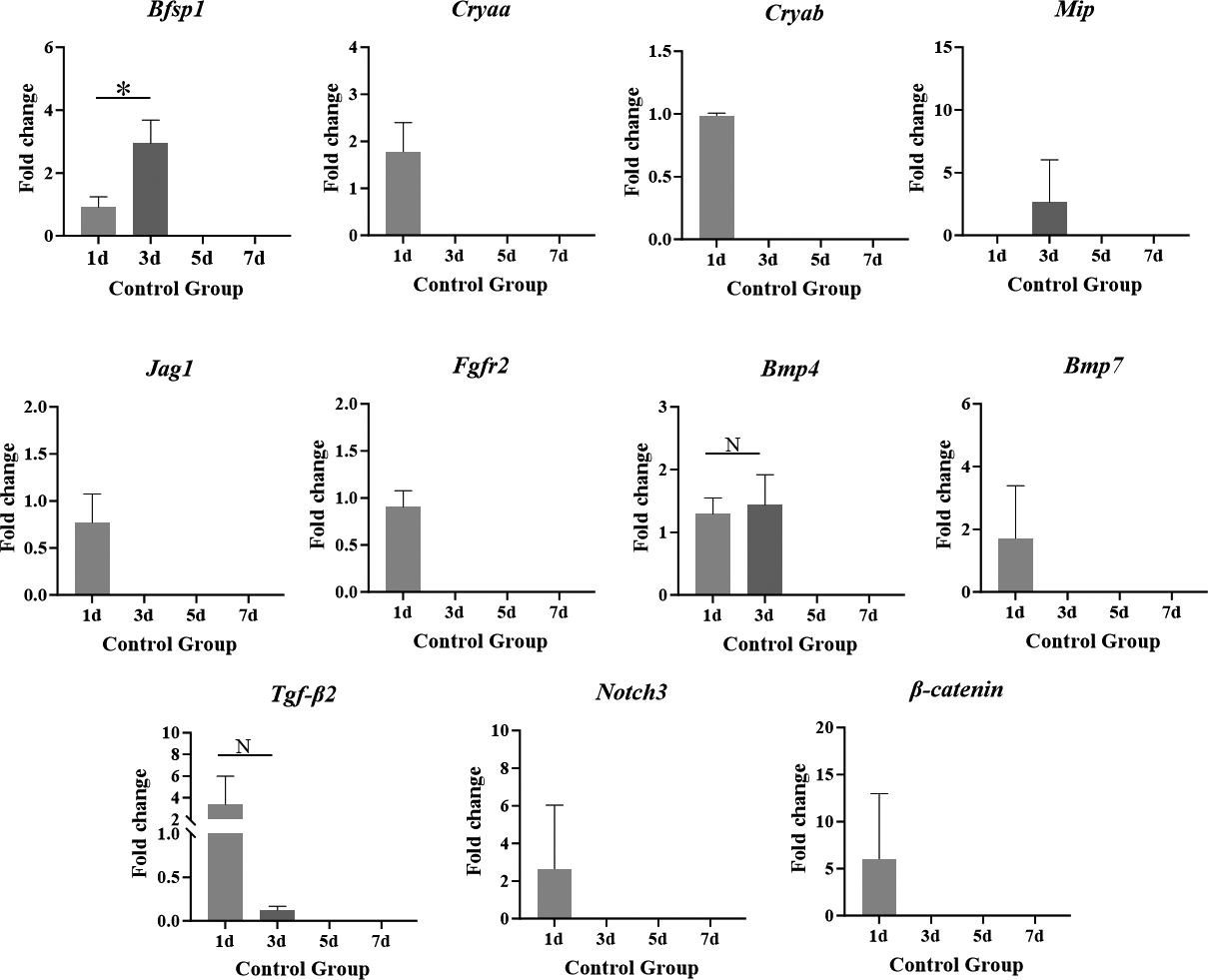

Supplement: Supplementary file 2 [file Image2.tif]

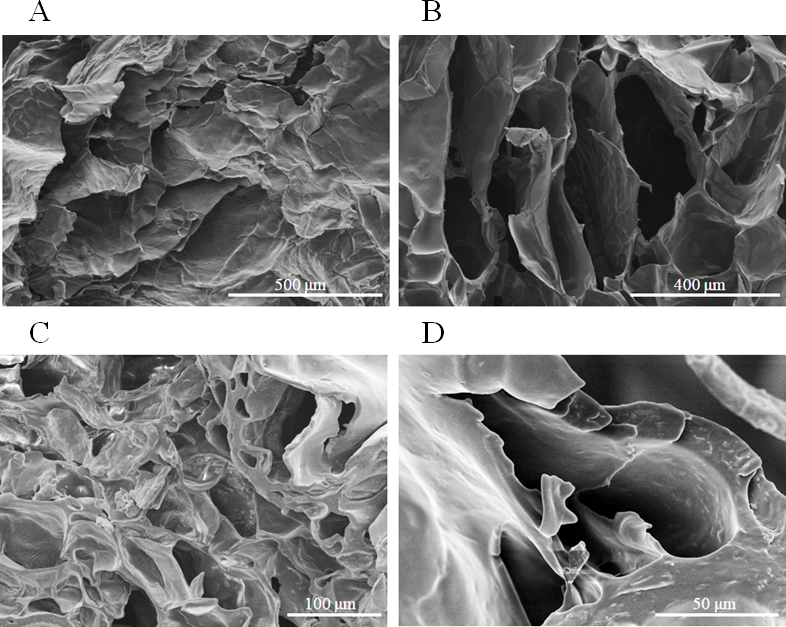

Supplement: Supplementary file 3 [file Image1.tif]
